# Potential caveats of putative microglia-specific markers for assessment of age-related cerebrovascular neuroinflammation

**DOI:** 10.1186/s12974-020-02019-5

**Published:** 2020-12-01

**Authors:** Pedram Honarpisheh, Juneyoung Lee, Anik Banerjee, Maria P. Blasco-Conesa, Parisa Honarpisheh, John d’Aigle, Abdullah A. Mamun, Rodney M. Ritzel, Anjali Chauhan, Bhanu P. Ganesh, Louise D. McCullough

**Affiliations:** 1 Department of Neurology, University of Texas John P. and Kathrine G. McGovern Medical School, Houston, TX USA; 2 UTHealth Graduate School of Biomedical Sciences, University of Texas MD Anderson Cancer Center, Houston, USA; 3 Department of Anesthesiology, Center for Shock, Trauma, and Anesthesiology Research, University of Maryland School of Medicine, Baltimore, MD USA

**Keywords:** Microglia, Neuroinflammation, Stroke, Cerebral amyloid angiopathy, Aging, Tmem119, P2RY12, CD45, Brain infiltrating myeloid cells

## Abstract

**Background:**

The ability to distinguish resident microglia from infiltrating myeloid cells by flow cytometry-based surface phenotyping is an important technique for examining age-related neuroinflammation. The most commonly used surface markers for the identification of microglia include CD45 (low-intermediate expression), CD11b, Tmem119, and P2RY12.

**Methods:**

In this study, we examined changes in expression levels of these putative microglia markers in in vivo animal models of stroke, cerebral amyloid angiopathy (CAA), and aging as well as in an ex vivo LPS-induced inflammation model.

**Results:**

We demonstrate that Tmem119 and P2RY12 expression is evident within both CD45^int^ and CD45^high^ myeloid populations in models of stroke, CAA, and aging. Interestingly, LPS stimulation of FACS-sorted adult microglia suggested that these brain-resident myeloid cells can upregulate CD45 and downregulate Tmem119 and P2RY12, making them indistinguishable from peripherally derived myeloid populations. Importantly, our findings show that these changes in the molecular signatures of microglia can occur without a contribution from the other brain-resident or peripherally sourced immune cells.

**Conclusion:**

We recommend future studies approach microglia identification by flow cytometry with caution, particularly in the absence of the use of a combination of markers validated for the specific neuroinflammation model of interest. The subpopulation of resident microglia residing within the “infiltrating myeloid” population, albeit small, may be functionally important in maintaining immune vigilance in the brain thus should not be overlooked in neuroimmunological studies.

**Supplementary Information:**

The online version contains supplementary material available at 10.1186/s12974-020-02019-5.

## Introduction

Resident microglia (MG) and infiltrating myeloid cells play a cooperative role in the initiation and resolution of inflammation after central nervous system (CNS) injuries [1]. Until recently, the ability to distinguish MG from infiltrating myeloid cells was constrained by the lack of cell-specific surface markers [1]. Inevitably, methods to distinguish MG from other CNS myeloid populations relied on morphological distinctions, generation of bone marrow chimeras, or *relative* expression of the common leukocyte antigen, also known as cluster of differentiation 45 (CD45) surface marker by flow cytometry [2–5]. CD45, a highly conserved receptor protein tyrosine phosphatase [6, 7], is used in various gating strategies to identify brain immune cell populations with flow cytometry and in single-cell profiling experiments [8]. Intermediate expression levels of CD45 combined with expression of CD11b (“CD45^int^CD11b^+^”) is often used to distinguish MG from peripherally sourced myeloid cells [5, 9–16]. However, this approach has an inherent limitation as CD45 expression itself may change in response to neuroinflammation in experimental models [17–19].

It has been suggested recently that MG possess a unique transcriptional signature that can be used in parallel with, or in lieu of, relative expression of CD45 for their reliable identification [9, 20–24]. Two of these putative markers are the trans-membrane protein 119 (Tmem119) and the purinergic receptor P2Y12 (P2RY12). Tmem119 is a cell-surface protein with unknown function in the brain [25], and P2RY12 is a well-studied purinergic receptor [26, 27]. Importantly, however, these markers have not been thoroughly validated in various animal models of age-related neuroinflammation [28, 29]. Additionally, downregulation of both Tmem119 and P2RY12 surface proteins has been recently demonstrated in experimental autoimmune encephalomyelitis (EAE) models [26, 27, 30–32]. Emerging evidence also supports the phenotypic plasticity of resident and infiltrating myeloid cells under chronic inflammatory conditions [33]. Quantifiable examination of Tmem119, P2RY12, and relative CD45 expression levels in neuroinflammation can provide valuable insight into the intricate relationship of brain-resident immune cells, infiltrating populations, and their interaction with the blood-brain barrier in neurological health and disease [30].

In this study, we hypothesized that a subpopulation of activated MG can upregulate CD45 and may be found within the CD45^high^ gate, which is conventionally classified as infiltrating myeloid cells [8, 14, 25, 31]. We tested our hypothesis using a mouse model of acute ischemic stroke (a surgical model) and CAA (a model of cerebrovascular degeneration). Our lab has recently shown that aging, a common clinical risk factor for both stroke and CAA [14, 32], independently alters the immunological response to stroke [14]. We also examined the independent effect of aging on the plasticity of CD45, Tmem119, and P2RY12 surface expressions within the brain myeloid compartment.

Our data show that the conventional gating strategy of CD45^int^ for MG vs. CD45^high^ for infiltrating cells can exclude a significant portion of Tmem119^+^P2RY12^+^ cells in three in vivo mouse models of ischemic stroke, CAA, and aging. Our ex vivo results demonstrate that CD45^int^ cells can indeed contribute to the CD45^high^ population upon inflammatory stimuli without any peripheral immune contribution. This suggests that CD45^high^Tmem119^+^P2RY12^+^ population is a heterogeneous mixture of activated resident and infiltrating myeloid cells. Importantly, this “hidden” subpopulation of resident MG within the CD45^high^CD11b^+^ gate may contain a functionally distinct MG subset compared to MG present within the conventional CD45^int^CD11b^+^ gate. Our results may have implications for understanding the role of activated MG versus infiltrating myeloid populations such as macrophages, dendritic cells, and monocyte-derived cells in neuroinflammation. This study particularly highlights the potential limitation of using *relative* expression of CD45 to identify activated MG in pre-clinical models of cerebrovascular injuries.

## Materials and methods

### Mice

C57BL/6 male mice were obtained from the National Institute on Aging (NIA). Young (2–4 months) and aged (16–22 months) were used in this study. Transgenic Swedish Dutch Iowa mice harboring the human APP gene (isoform 770) with the Swedish (K670N/M671L), Dutch (E693Q), and Iowa (D694N) mutations under control of the mouse Thy1.2 promoter were used at a pre-symptomatic time point of 1–3 months, and a peak symptomatic time point of 12–15 months, here referred to as “CAA” mice [28, 29, 33, 34]. Cognitive deficits begin at 3–4 months of age in the Tg-SwDI mice, as detected by Barnes maze [35–37]. All animals were group-housed in Tecniplast individually ventilated cage (IVC) racks, fed a commercially available irradiated, balanced mouse diet (no. 5058, LabDiet, St Louis, MO), and provided corncob bedding. Rooms were maintained at 70–73 °F and under a 12:12-h light:dark cycle. All animals were maintained specific pathogen-free (see Supplementary Material for list of monitored pathogens). Animal procedures were performed at an AAALAC accredited facility and were approved by the Animal Welfare Committee at the University of Texas Health Science Center at Houston, TX, USA.

### Middle cerebral artery occlusion (MCAO)

Transient focal ischemia was induced under isoflurane anesthesia in young mice (12–16 weeks) for 60 min by occlusion of the right middle cerebral artery [38]. Body temperature was maintained at 37.0 ± 1.0 °C throughout the surgery by an automated temperature control feedback system (TC1000, mouse, CWE Inc., USA). A midline ventral neck incision was made, and unilateral MCAO was performed by inserting a Doccol monofilament (Doccol Corp., Redlands, CA, USA) into the right internal carotid. Cerebral blood flow (CBF) was measured by Laser Doppler flowmetry (Moor Instruments Ltd., Devor, England). CBF was measured before ischemia, during ischemia, and at the time of reperfusion. One hour after ischemia, animals were anesthetized again and reperfusion was established by the withdrawal of the monofilament. Animals were then placed in a recovery cage and were euthanized 72 h after reperfusion. Sham controls were subjected to the same procedure without the introduction of the suture into the middle cerebral artery. Animals were randomly assigned into stroke and sham surgery groups and single-housed in their recovery cages for the first 2 h after surgery. Sham and stroke mice were then housed together in their home cages to minimize the detrimental effects of social isolation [39]. All mice were selected for sham or stroke (MCAO) surgery in a randomized manner, and all analyses were performed blinded to surgical conditions.

### Flow cytometry (brain and blood)

A previously published brain single-cell suspension protocol was used [14, 40]. In brief, mice were euthanized by avertin injection. The blood was drawn by cardiac puncture with heparinized needles. Red blood cell lysis was achieved by two consecutive 10-min incubations with Tris–ammonium chloride (Stem Cell Technologies). Mice were transcardially perfused with a 20-ml cold, sterile PBS prior to aseptic removal of brain tissues. The brain tissue was placed in complete Roswell Park Memorial Institute 1640 (Lonza) medium and then mechanically and enzymatically digested in Collagenase/Dispase (1 mg/mL) and DNase (10 mg/mL; Roche Diagnostics) for 45 min at 37 °C with gentle shaking (100 RPM). The cell suspension was filtered through a 70-μm filter. Leukocytes were harvested from the interphase of 70–30% Percoll gradients for the brain tissue. Cells were washed and blocked with mouse Fc Block (BioLegend, Lot: B298973) before staining with primary antibody-conjugated fluorophores: CD45-eF450 (eBioscience, Cat#: 48-0451-82, Lot: 2005853), CD11b-APC (BioLegend, Cat#: 101212, Lot: B279418), Ly6C-PerCP-Cy5.5 (BioLegend, Cat#: 128011, Lot: 292026), Tmem119-PE-Cy7 (eBioscience, Cat#: 25-6119-82, Lot: 2210260), P2RY12-PE (BioLegend, Cat#: 848003, B298459), and MHCII-APC-Fire750 (BioLegend, Cat#: 107652, Lot: B301025) pre-conjugated antibodies and Zombie Aqua (BioLegend, Cat#: 423102, Lot: B300004). Cell isolation, Percoll gradient, and immunostaining steps were carried out at once for both controls and injury models to minimize experimental variabilities, i.e., all sham and stroke samples were processed together, all pre-CAA and CAA samples were processed together, and all naïve young and aged samples were processed together. Data were acquired on Cytoflex-S (Beckman Coulter) or BD FACSMelody cytometers and analyzed using FlowJo (Treestar Inc.). No less than 300,000 events were recorded for each sample, and absolute cell counts have been included in Supplementary Table 1. Tissue-matched fluorescence minus one (FMO) and unstained controls were used to aid in the gating strategy (Supplementary Fig 1). t-distributed stochastic neighbor embedding (tSNE) plots were generated in FlowJo using DownSample plug-in (3000 cells per sample for each study group) followed by tSNE algorithm on all compensated parameters (except viability) at 1000 iterations, perplexity of 30, learning rate of 5040, and Barnes-Hut gradient algorithm.

### Cell sorting

Single-cell suspension and surface staining were performed as described above. After viability and single-cell selections, MG, gated as CD11b^+^CD45^int^, were sorted under a sterile hood from the single-cell suspension prepared from naïve young male brains (full brains, *n* = 10) using BD FACSMelody. Each sorted sample was then split in half (by volume) into a pair of tubes (traced for analysis) and either incubated with lipopolysaccharide (LPS) or with control media for 12 h under a sterile cell-culture environment. Cells were then extensively washed with PBS, stained for surface markers and viability, and analyzed by flow cytometry.

### Ex vivo LPS treatments

Brain immune cell isolation was performed by optimized enzymatic digested followed by Percoll gradient protocol [14, 41]. For ex vivo studies, LPS (at 100 ng/mL concentration, from *Escherichia coli* O111:B4, purified by phenol extraction, Millipore Sigma) was added to RPMI (containing 10% heat-inactivated fetal bovine serum, Sigma Aldrich, Cat: 12106C, ≤ 10 EU/mL endotoxin) and incubated at the sterile cell-culture environment at 37 °C for 12 h. The brain tissue was harvested after cardiac perfusion with PBS to eliminate the blood from the brain tissue. There is a possibility that PBS-perfused samples contain some blood-sourced immune cells prior to ex vivo LPS challenge. Thus, we performed digestion and Percoll gradient-based separation of microglia at which point, each individual sample was split in half (by volume) into a pair of LPS-treated and control. Splitting each individual brain sample into a pair of LPS and control after digestion and gradient-based isolation steps allowed us to verify that control tubes had significantly lower CD45^high^CD11b^+^ cells when compared to LPS-treated cells from the same brain after identical single-cell suspension preparation (see Fig. 4a, c for the schematics).

### Statistical analysis

**Fig. 1:**
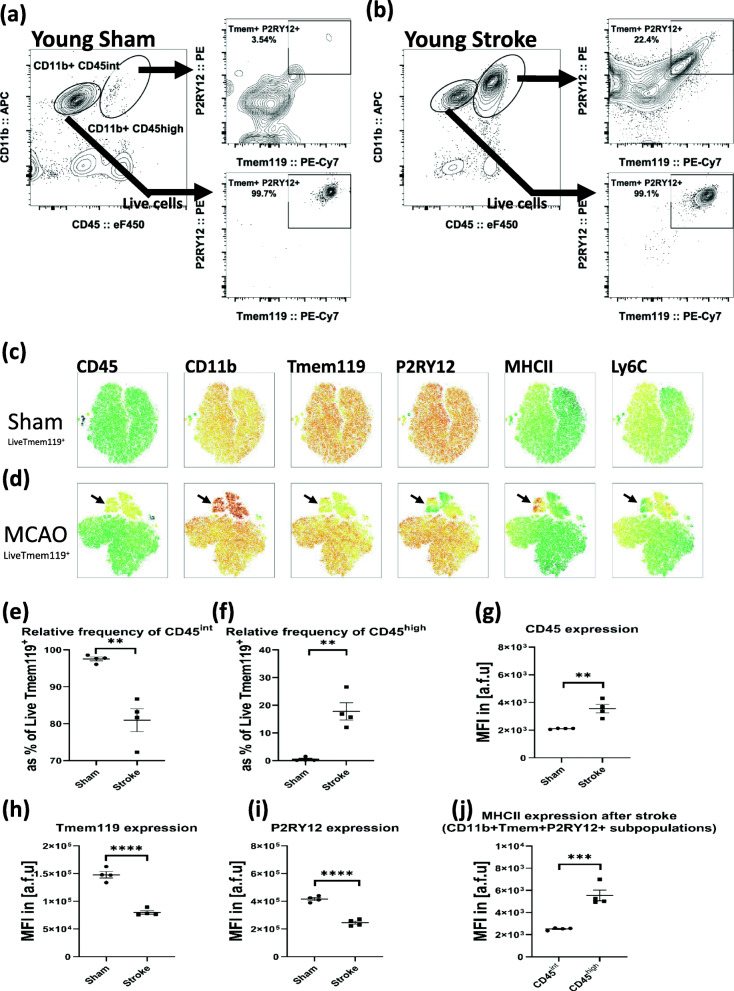
Examination of brain myeloid populations after a 60-min MCAO reveals a significant population of Tmem119^+^P2RY12^+^ cells with relatively higher MHC-II expression resides within the CD45^high^CD11b^+^ gate. **a** Ipsilateral hemisphere from the sham brain (gated on live cells) myeloid populations shows that nearly all CD45^int^CD11b^+^ cells are double-positive Tmem119^+^P2RY12^+^ while no significant subpopulation of Tmem119^+^P2RY12^+^ cells resides within the CD45^high^CD11b^+^ gate. **b** Ipsilateral hemisphere from stroke brain shows that nearly all CD45^int^CD11b^+^ cells are double-positive Tmem119^+^P2RY12^+^ and show a significant subpopulation of Tmem119^+^P2RY12^+^ cells within the CD45^high^CD11b^+^ gate. **c** tSNE plots for the global examination of Live Tmem119^+^ pool in the sham brain. **d** tSNE plots for the global examination of Live Tmem119^+^ pool in the ischemic brain. The black arrow shows the CD45^high^ cluster which can be identified for other surface markers in MCAO tSNE plots. **e** Relative frequency of CD45^int^ population as a percentage of Live Tmem119^+^ cells is significantly lower after MCAO. **f** Relative frequency of CD45^high^ population as a percentage of Live Tmem119^+^ cells is significantly increased after MCAO. **g** Median fluorescence intensity (MFI) of CD45, as a measure of surface expression, in Live Tmem119^+^ cells is significantly increased after MCAO. **h** Surface expression of Tmem119 in Live Tmem119^+^ cells is significantly decreased after MCAO. **i** Surface expression of P2RY12 in Live Tmem119^+^ cells is significantly decreased after MCAO. **j** Surface expression of MHCII in CD45^int^ versus CD45^high^ subpopulations of CD11b^+^Tmem119^+^P2RY12^+^ cells shows significantly higher MHCII expression in the CD45^high^ subpopulation after MCAO. (*n* = 4/gp, unpaired student *t* test, **p < 0.01, ***p < 0.001, ****p < 0.0001)

**Fig. 2:**
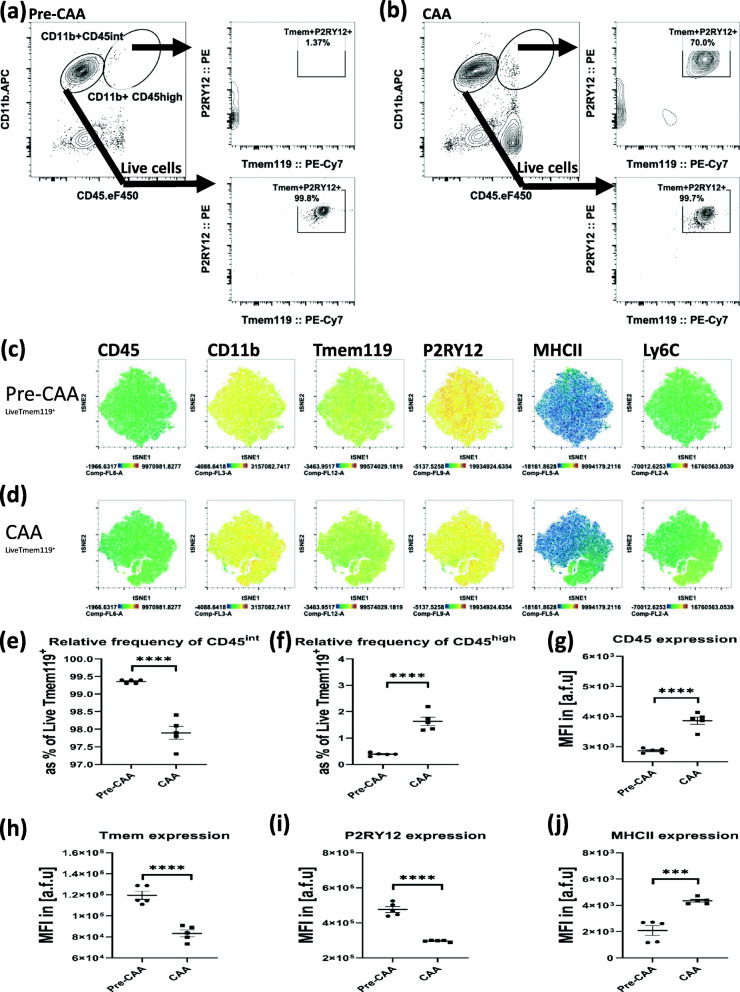
Examination of brain myeloid populations in pre-symptomatic Tg-SwDI (“Pre-CAA”) versus symptomatic Tg-SwDI (“CAA”) reveals that the CD45^high^CD11b^+^cells are predominantly Tmem119^+^P2RY12^+^. **a** Pre-CAA brain (gated on live cells) myeloid populations show that nearly all CD11b^+^CD45^int^ cells are double-positive Tmem119^+^P2RY12^+^ while no subpopulation of Tmem119^+^P2RY12^+^ cells resides within the CD11b^+^CD45^high^ gate. **b** CAA brain myeloid populations show that nearly all CD11b+ CD45^int^ cells are double-positive Tmem119^+^P2RY12^+^ and show a significant subpopulation of Tmem119^+^P2RY12^+^ cells within the CD11b^+^CD45^high^ gate. **c** tSNE plots for the global examination of Live Tmem119^+^ pool in Pre-CAA brain. **d** tSNE plots for the global examination of Live Tmem119^+^ pool in the CAA brain. **e** Relative frequency of CD45^int^ population as a percentage of Live Tmem119^+^ cells is significantly lower in CAA. **f** Relative frequency of CD45^high^ population as a percentage of Live Tmem119^+^ cells is significantly higher in CAA. **g** Median fluorescence intensity (MFI) of CD45, as a measure of surface expression, in Live Tmem119^+^ cells is significantly higher in CAA. **h** Surface expression of Tmem119 in Live Tmem119^+^ cells is significantly lower in CAA. **i** Surface expression of P2RY12 in Live Tmem119^+^ cells is significantly lower in CAA. **j** Surface expression of MHCII in Live Tmem119^+^ cells is significantly higher in CAA. (*n* = 4/gp, unpaired student *t* test, ***p < 0.001, ****p < 0.0001)

**Fig. 3:**
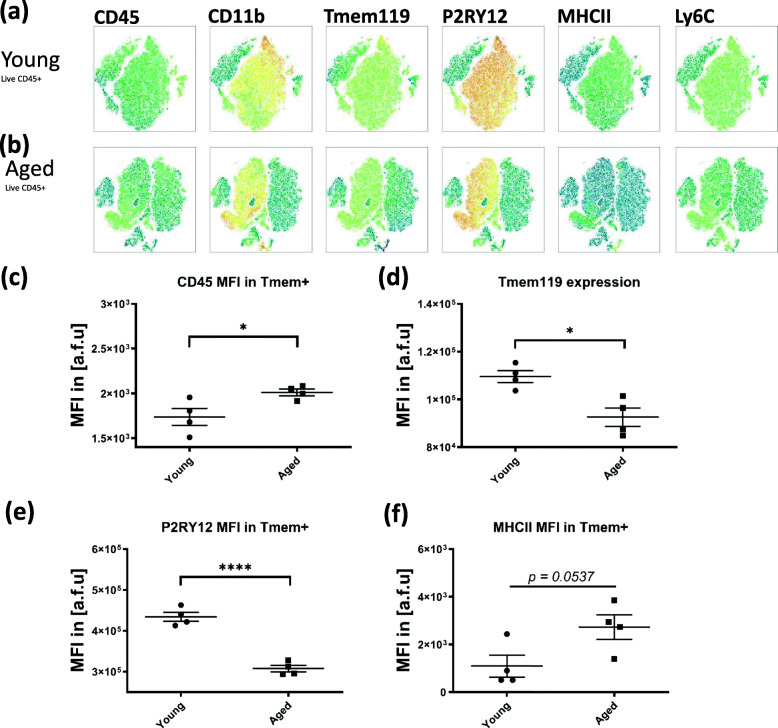
Examination of brain myeloid populations in young (2–4 months) and aged (16–22 months) naïve brains shows a significant increase in CD45 (**a**), decrease in Tmem119 (**b**), and decrease in P2RY12 (**c**) expressions with aging in Live Tmem119^+^ cells. (*n* = 4/gp, unpaired student *t* test, *p < 0.05, ****p < 0.0001)

**Fig. 4:**
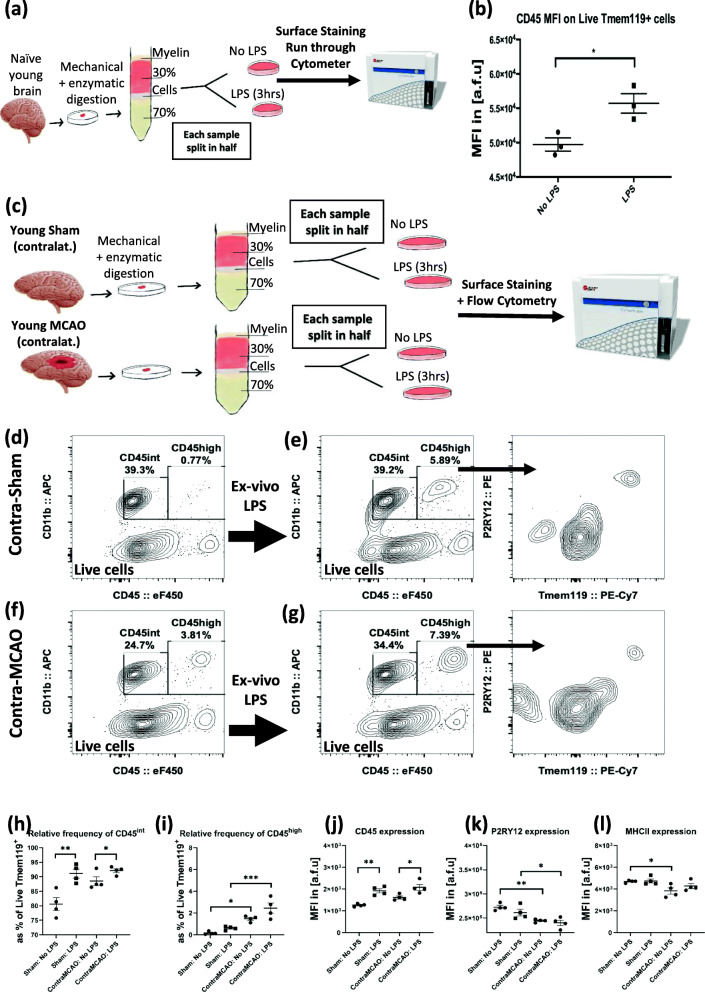
Ex vivo LPS-induced inflammation of brain immune cells shows increased CD45 expression within LiveTmem119^+^ cells. The post-Percoll brain cells *from the same naïve young brain hemispheres* were incubated with or without LPS at 37C for 3 h prior to staining and flow cytometric analysis (**a**). Examination of Live Tmem119^+^ cells from naïve young brain shows that ex vivo LPS-induced inflammation leads to a significant upregulation of CD45 expression (**b**). To determine whether stroke will reduce the ability of brain immune cells to upregulate their CD45 expression without any peripheral contribution of the infiltrating immune cells, ex vivo LPS-induced inflammation studies were carried out using contralateral hemispheres of sham and MCAO brains (**c**–**l**). No significant population of CD11b^+^CD45^high^ cells was present in the young sham brain without LPS treatment (**d**), but a significant population of CD11b^+^CD45^high^ is found in the young sham contralateral brain with LPS treatment (**e**). Results show a significant increase in CD11b^+^CD45^high^ population when comparing LPS-treated post-Percoll brain cells with untreated cells from the same contralateral hemisphere of stroke brains (“Contra-MCAO”, **f**, **g**). The analysis shows a significant upregulation of CD45 expression with LPS stimulation in both sham and MCAO assays (**j**). (*n* = 4/gp, a one-way ANOVA with Sidak’s multiple comparisons test, *p < 0.05, **p < 0.01, ***p < 0.001)

**Fig. 5:**
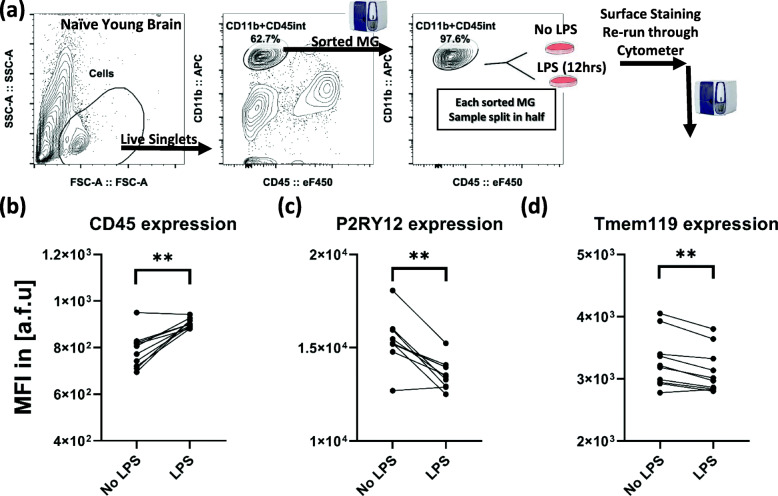
Ex vivo LPS stimulation of sorted MG (identified as CD45^int^CD11b^+^ in naïve young brain) increases CD45 and decreases P2RY12 and Tmem119 surface expressions. Gating strategy used for sorting Live CD45^int^CD11b^+^cells for 12-h ex vivo LPS stimulation (**a**). CD45 expression on sorted MG significantly increases after ex vivo LPS stimulation for 12 h (**b**). P2RY12 and Tmem119 expressions on sorted MG significantly decrease after ex vivo LPS stimulation for 12 h (**c**, **d**). (*n* = 10/gp, Wilcoxon matched-pairs signed-rank test (non-parametric *t* test), **p < 0.01)

Statistical analysis was performed using unpaired *t* test for sham vs. MCAO (Fig. 1), pre-CAA vs. CAA (Fig. 2), and young vs. aged (Fig. 3) brain flow cytometry data. Ex vivo LPS experimental data from the contralateral hemisphere of sham vs. MCAO brains (Fig. 4) were analyzed by a one-way ANOVA with post hoc analysis with all related *p* values adjusted by Sidak’s methods for multiple comparisons. Ex vivo LPS stimulation of sorted MG experimental data was analyzed by Wilcoxon matched-pairs signed-rank test because each pair of non-LPS vs. LPS was traced throughout the experiment; thus, we were able to perform a pair-wise analysis and represent the data accordingly (Fig. 5). The statistical significance was considered at *p* < 0.05 and *p < 0.05, **p < 0.01, ***p < 0.001, and ****p < 0.0001 convention was used in the presented figures. All statistical analyses were performed with GraphPad Prism 7.

## Results

### The conventional infiltrating myeloid gate (CD45^high^CD11b^+^) contains a mixture of resident and infiltrating immune cells in acute ischemic stroke

Young male mice underwent a 60-min reversible MCAO and were euthanized on post-stroke day 3. The examination of brain myeloid populations reveals that a subpopulation of Tmem119^+^P2RY12^+^ cells resides within the CD45^high^CD11b^+^ gate (Fig. 1). Absolute numbers of Tmem119^+^ cells and P2RY12^+^ cells were almost identical in individual samples, and we verified that all Tmem119^+^ cells were indeed P2RY12^+^ (Supplementary Fig 2); thus, statistical analyses were carried out based on Tmem119^+^ counts as a proxy for Tmem119^+^P2RY12^+^ counts. The brains from sham mice did not show a significant population of CD45^high^CD11b^+^ cells (Fig. 1a), while the brains from MCAO mice had a significant CD45^high^CD11b^+^ population (Fig. 1b). All CD45^int^CD11b^+^ cells in both sham and stroke were Tmem119^+^P2RY12^+^ (Fig. 1a, b, bottom panels). Interestingly, a significant subpopulation of CD45^high^CD11b^+^ population was Tmem119^+^P2RY12^+^ in stroke but not in sham brains (Fig. 1a, b, top panels). Multi-dimensional analysis of flow cytometry data revealed the heterogeneity of CD45^high^ cells when comparing sham and MCAO brains (Fig. 1c, d). Specifically, the CD45^high^ cluster in the MCAO tSNE plots (marked by a black arrow in Fig. 1d) showed a uniformly high expression of CD11b while all other examined surface markers, i.e., Tmem119, P2RY12, MHCII, and Ly6C showed significant heterogeneity of expression levels (Fig. 1d). The statistical analysis showed a significant increase in the relative frequency of the CD45^high^ subpopulation as a percent of Tmem119^+^ cells after stroke which was associated with a significant decrease in Tmem119 and P2RY12 expressions (Fig. 1f–i). MHC-II surface expression of CD45^high^CD11b^+^Tmem119^+^P2RY12^+^ subpopulation was significantly higher than CD45^int^CD11b^+^Tmem119^+^P2RY12^+^ (Fig. 1j), suggesting a higher activation state of these CD45^high^ cells after stroke [42, 43]. We then analyzed CD45^high^CD11b^+^ myeloid subpopulations that were double-negative or double-positive for Tmem119 and P2RY12 for expression of Ly6C, a marker of bone marrow-derived cells [44]. We found that CD45^high^CD11b^+^Tmem119^+^P2RY12^+^ were predominantly Ly6C^(-)^ while CD45^high^CD11b^+^Tmem119^(-)^P2RY12^(-)^ were predominantly Ly6C^+^, suggesting their peripheral origin (Supplementary Fig 2b). Confirmatory analysis of blood samples from both sham and stroke mice did not show any significant population of Tmem119^+^P2RY12^+^ cells (Supplementary Fig 3). These findings highlight the heterogeneity of the CD45^high^CD11b^+^ brain myeloid population after stroke and suggest partial contributions from both resident and peripherally sourced myeloid cells, when examining Tmem119, P2RY12, and Ly6C markers. Importantly, a small subpopulation of resident MG that express both Tmem119 and P2RY12 may fall within the CD45^high^CD11b^+^ gate that is functionally distinct after stroke when compared to the large subpopulation of resident MG identified as CD45^int^CD11b^+^.

### CD45^high^CD11b^+^ brain myeloid cells are predominantly Tmem119^+^P2RY12^+^ in animal models of CAA

Next, we addressed whether CD45^high^CD11b^+^Tmem119^+^P2RY12^+^ cells exist in transgenic mouse models of cerebrovascular degeneration. To this end, we used pre-symptomatic (1–3 months, “Pre-CAA”) and symptomatic (12–15 months, “CAA”) Tg-SwDI mice, which begin developing symptoms around 4 months and exhibit peak CAA symptoms at around 12 months. Examination of pre-symptomatic CAA mice showed that the CD45^high^CD11b^+^ gate did not contain any significant Tmem119^+^P2RY12^+^ cells (Fig. 2a, c), while in symptomatic CAA mice, the CD45^high^CD11b^+^ cells were predominantly Tmem119^+^P2RY12^+^ (Fig. 2b, d). Examination of Tmem119^+^ cells in CAA compared to pre-CAA brain showed that both the relative frequency of CD45^high^ cells and surface expression of CD45 significantly increased in the CAA brain (Fig. 2e-g), which was associated with a significant decrease in Tmem119 and P2RY12 and a significant increase in MHC-II expression (Fig. 2h–j). Consistent with previous reports using RNA sequencing [45], our flow cytometric comparison of young and aged naïve brain showed that CD45 expression by Tmem119^+^ cells significantly increased with aging and is associated with a significant decrease in Tmem119 and P2RY12 surface protein expressions (Fig. 3c–e).

### Ex vivo LPS treatment induces similar phenotypic changes in FACS-sorted MG

Ex vivo LPS-induced inflammation models were used to determine whether CD45^int^CD11b^+^Tmem119^+^P2RY12^+^ cells from the brain can upregulate their CD45 expression, independently of contributions from the peripheral immune system. Brain immune cell isolation was performed by optimized enzymatic digestion followed by Percoll gradient protocol (Fig. 4a) [14, 41]. After the Percoll gradient step for myelin removal, the post-Percoll brain cells from the same brain hemispheres were incubated with or without LPS for 3 h prior to surface marker staining and analysis by flow cytometry (Fig. 4a, c). Examination of Tmem119^+^ cells from naïve young brain showed that ex vivo LPS-induced inflammation led to a significant upregulation of CD45 expression (Fig. 4b). To determine if stroke could affect the ability of CD45^int^Tmem119^+^P2RY12^+^ cells to upregulate their CD45 expression without any peripheral contribution of the infiltrating immune cells, ex vivo LPS-induced inflammation studies were carried out using the contralateral hemispheres of sham and MCAO young brains (Fig. 4c–l). Data showed that regardless of a stroke status, post-Percoll brain Tmem119^+^ cells acutely upregulate expression of CD45 in response to an inflammatory LPS stimulus (Fig. 4e, j). Minimal CD45^high^CD11b^+^ cells were present in the sham brain without LPS treatment while a significant population of CD45^high^CD11b^+^ was found in the sham brain after ex vivo LPS treatment (Fig. 4e, f). Furthermore, these post-LPS CD45^high^CD11b^+^ cells contain a mixed population of Tmem119^+^P2RY12^+^ and Tmem119^(-)^P2RY12^(-)^ cells (Fig. 4e). Results showed that cells from the same contralateral hemisphere from stroke mice had a significant increase in the CD45^high^CD11b^+^ population when treated with ex vivo LPS (Fig. 4f, g). Analysis demonstrated a significant upregulation of CD45 expression with LPS stimulation in both sham and MCAO assays (Fig. 4j). These results showed that CD45^int^ MG can indeed upregulate expression of CD45. However, the treated samples used in these experiments contained other non-MG CNS cells. Next, we asked whether the in vivo and ex vivo post-LPS increase in CD45 expression by MG is dependent on other CNS cells. To examine this, MG were sorted from naïve young brain; then, each sample was split in half (by volume) before incubation with either LPS or control media for 12 h (Fig. 5a). Consistently, post-LPS analysis of sorted MG showed a significant increase in CD45 expression by MG, which was also associated with significant downregulation of Tmem119 and P2RY12 surface expression (Fig. 5b–d).

Taken together, our data show that resident CD45^int^Tmem119^+^P2RY12^+^ cells are capable of upregulating their CD45 while downregulating Tmem119 and P2RY12 expression in neuroinflammatory conditions such as stroke, CAA, or aging and may reside within the CD45^high^CD11b^+^ gate, conventionally designated as “infiltrating myeloid cells.” Importantly, the CD45^high^ subpopulation of MG within the CD45^high^CD11b^+^ gate, albeit small, may be functionally more important in maintaining immune vigilance in the brain, as suggested by higher MHC-II expression levels [42, 43]. Recent studies have also shown that the presence of this small CD45^high^ MG may persist until post-stroke day 14 [16].

## Discussion

Prior to recent evidence suggesting that MG can be reliably identified using MG-specific surface markers [9, 20–23], *relative* expression of the CD45 surface marker by flow cytometry was among the few available methods to distinguish MG from infiltrating myeloid cells [2–4]. However, this approach is inherently limited by the changes in CD45 expression in the context of specific CNS pathologies, which has been reported by our lab and others [5, 46, 47]. In this study, we used Tmem119 and P2RY12 along with a conventional combination of CD45, CD11b, and Ly6C myeloid markers in models of stroke, CAA, and aging to determine whether Tmem119^+^P2RY12^+^ cells appear within the CD45^high^CD11b^+^ gate, often referred to as the “infiltrating myeloid” gate. Further, we demonstrated that CD45^int^Tmem119^+^P2RY12^+^ can upregulate CD45 and downregulate Tmem119 and P2RY12 expression in response to an inflammatory stimulus even without any direct contributions from the peripheral immune system or other CNS cells.

Our data suggest that microglia continuously sense the environment and actively respond to both acute and chronic brain injuries by altering their surface molecular signatures. Specifically, brain resident CD45^int^CD11b^+^Tmem119^+^P2RY12^+^ cells can alter their surface expression patterns after stroke, in symptomatic CAA, or with aging and can appear in the CD45^high^ gate in flow cytometry studies. Although Tmem119 and P2RY12 are well-characterized microglia markers, our data does *not* rule out the possibility that peripherally sourced myeloid cells may possess the capacity to express Tmem119 and P2RY12 when in the CNS environment and thus appear as CD45^high^CD11b^+^Tmem119^+^P2RY12^+^ cells. In fact, recent evidence suggests that Tmem119^+^ MG-like cells can be found in the brain based on ontogeny and under the influence of CNS milieu [48]. Others have found that peripheral macrophages express Tmem119 and P2RY12 during development [49]. Consistent with recent studies in pre-clinical models of Alzheimer’s disease (AD) and EAE [48, 50–52], we also report both Tmem119 and P2RY12 expression are decreased after acute ischemic stroke, after the development of symptoms in CAA models, and in ex vivo models of inflammation for sorted MG. Expression of Ly6C, a marker of bone marrow-derived myeloid cells (similar to CCR 2[44]), showed that the majority of CD45^high^ myeloid cells that were double negative for Tmem119 and P2RY12 were indeed Ly6C^+^, suggesting their peripheral origin. Conversely, the majority of Tmem119 and P2RY12 double-positive cells in the CD45^high^ gate were Ly6C^(-)^, suggesting their brain-resident origin. Intriguingly, a small percentage of CD45^high^Tmem119^+^P2RY12^+^ were also Ly6C^+^, supporting other recent studies [48, 49] that peripherally sourced myeloid cells may possess the capacity to express Tmem119 and P2RY12 as well as the ability to downregulate Ly6C, making them less distinguishable from the brain-resident cells. These findings are consistent with evidence that bone marrow-derived monocytes may downregulate their Ly6C expression and establish a long-term presence in the injured brain [53]. We also reported that no significant population of Tmem119^+^P2RY12^+^ cells are present in the peripheral blood of sham or MCAO mice 3 days after stroke. This data supports our hypothesis; however, it does not eliminate the possibility that blood-sourced myeloid cells may enter the brain earlier during the injury and start expressing some level of Tmem119 or P2RY12 under the influence of the CNS milieu. Future experiments that include the analyses of the brain tissue, blood, and brain lymphatic samples after a stroke at multiple time points will be of great value in clarifying potential sources of Tmem119^+^P2RY12^+^ cells following ischemia.

Our findings may have implications in previous reports focused on the roles of activated MG versus infiltrating non-microglia myeloid cell populations [5, 9–13, 54–56] and highlight the diversity of brain myeloid compartment in neuroinflammation [57]. Future studies utilizing bone marrow chimeras and Tmem119 or P2RY12 deletion in MG can elucidate the mechanistic effects of changes in MG surface markers in neuroinflammation.

### Conclusion

In conclusion, we recommend that future studies should approach the identification of MG by flow cytometry with caution, particularly in the absence of a combination of markers validated for the specific neuroinflammation model of interest.

## Supplementary Information

**Additional file 1: Table S1.** Absolute cell counts for brain flow preps from automated volumetric data obtained from cytometers (Cytoflex S for Main Figs. 1 and 2 and BD FACSMelody for Fig. 5) for MCAO **(a)**, CAA **(b)**, and ex-vivo LPS on sorted MG **(c)** experiments.

**Additional file 2: Figure S1.** Brain gating strategy for Live Tmem119^+^ cells using fluorescence minus one control for positive gating. **Figure S2.** Evaluation of Live Tmem119^+^ population for P2RY12 indicates that all Tmem119^+^ cells are P2RY12^+^, as well (top plots) and vice versa (bottom plots) **(a)**. Within the CD45^high^CD11b^+^population, a small percentage of double positive for Tmem119 and P2RY12 were Ly6C^+^ while the Tmem119^(-)^P2RY12^(-)^ were indeed predominantly Ly6C^+^, suggesting their peripheral origin **(b)**. **Figure S3.** Blood Live cells from sham or stroke mice do not contain any significant Tmem119^+^P2RY12^+^ cells.

## Data Availability

We agree that upon request, all the repeatability and reproducibility data files will be provided.

## Abbreviations

MG: Microglia
CNS: Central nervous system
CD: Cluster of differentiation
Tmem119: Trans-membrane protein 119
P2RY12: Purinergic receptor P2Y12
CAA: Cerebral amyloid angiopathy
MCAO: Middle cerebral artery occlusion
LPS: Lipopolysaccharide
tSNE: t-distributed stochastic neighbor embedding
AD: Alzheimer’s disease
EAE: Experimental autoimmune encephalomyelitis

## References

[1] Greenhalgh AD, Passos Dos Santos R, Zarruk JG, Salmon CK, Kroner A, David S (2016). Arginase-1 is expressed exclusively by infiltrating myeloid cells in CNS injury and disease. Brain Behav Immun.

[2] Ford AL, Goodsall AL, Hickey WF, Sedgwick JD (1995). Normal adult ramified microglia separated from other central nervous system macrophages by flow cytometric sorting. Phenotypic differences defined and direct ex vivo antigen presentation to myelin basic protein-reactive CD4+ T cells compared. J Immunol.

[3] Lassmann H, Schmied M, Vass K, Hickey WF (1993). Bone marrow derived elements and resident microglia in brain inflammation. Glia.

[4] Ajami B, Bennett JL, Krieger C, Tetzlaff W, Rossi FMV (2007). Local self-renewal can sustain CNS microglia maintenance and function throughout adult life. Nat Neurosci.

[5] Ritzel RM, Patel AR, Grenier JM, Crapser J, Verma R, Jellison ER (2015). Functional differences between microglia and monocytes after ischemic stroke. J Neuroinflammation.

[6] Rheinländer A, Schraven B, Bommhardt U (2018). CD45 in human physiology and clinical medicine. Immunol Lett.

[7] Hermiston ML, Xu Z, Weiss A (2003). CD45: a critical regulator of signaling thresholds in immune cells. Annu Rev Immunol.

[8] Jordão MJC, Sankowski R, Brendecke SM, Sagar LG, Tai Y-H (2019). Single-cell profiling identifies myeloid cell subsets with distinct fates during neuroinflammation. Science.

[9] Butovsky O, Jedrychowski MP, Moore CS, Cialic R, Lanser AJ, Gabriely G (2014). Identification of a unique TGF-β-dependent molecular and functional signature in microglia. Nat Neurosci.

[10] Chauhan A, Al Mamun A, Spiegel G, Harris N, Zhu L, McCullough LD (2018). Splenectomy protects aged mice from injury after experimental stroke. Neurobiol Aging.

[11] Kolter J, Kierdorf K, Henneke P (2020). Origin and differentiation of nerve-associated macrophages. J Immunol.

[12] Felger JC, Abe T, Kaunzner UW, Gottfried-Blackmore A, Gal-Toth J, McEwen BS (2010). Brain dendritic cells in ischemic stroke: time course, activation state, and origin. Brain Behav Immun.

[13] Fumagalli S, Perego C, Ortolano F, De Simoni M-G (2013). CX3CR1 deficiency induces an early protective inflammatory environment in ischemic mice. Glia.

[14] Ritzel RM, Lai Y-J, Crapser JD, Patel AR, Schrecengost A, Grenier JM (2018). Aging alters the immunological response to ischemic stroke. Acta Neuropathol.

[15] Martin E, El-Behi M, Fontaine B, Delarasse C. Analysis of microglia and monocyte-derived macrophages from the central nervous system by flow cytometry. J Vis Exp. 2017; Available from: http://www.ncbi.nlm.nih.gov/pubmed/28671658. 10.3791/55781 PMC5608497 28671658

[16] Weitbrecht L, Berchtold D, Zhang T, Jagdmann S, Dames C, Winek K, et al. CD4+ T cells promote delayed B cell responses in the ischemic brain after experimental stroke. Brain Behav Immun. 2020; Available from: http://www.ncbi.nlm.nih.gov/pubmed/33002634. 10.1016/j.bbi.2020.09.029 33002634

[17] Finneran DJ, Morgan D, Gordon MN, Nash KR (2019). CNS-wide over expression of fractalkine improves cognitive functioning in a tauopathy model. J Neuroimmune Pharmacol.

[18] Bennett ML, Bennett FC, Liddelow SA, Ajami B, Zamanian JL, Fernhoff NB (2016). New tools for studying microglia in the mouse and human CNS. Proc Natl Acad Sci U S A.

[19] Stein VM, Baumgärtner W, Schröder S, Zurbriggen A, Vandevelde M, Tipold A (2007). Differential expression of CD45 on canine microglial cells. J Vet Med A Physiol Pathol Clin Med.

[20] Grabert K, Michoel T, Karavolos MH, Clohisey S, Baillie JK, Stevens MP (2016). Microglial brain region-dependent diversity and selective regional sensitivities to aging. Nat Neurosci.

[21] Hickman SE, Kingery ND, Ohsumi TK, Borowsky ML, Wang L, Means TK (2013). The microglial sensome revealed by direct RNA sequencing. Nat Neurosci.

[22] Wes PD, Holtman IR, Boddeke EWGM, Möller T, Eggen BJL (2016). Next generation transcriptomics and genomics elucidate biological complexity of microglia in health and disease. Glia.

[23] Guerreiro R, Wojtas A, Bras J, Carrasquillo M, Rogaeva E, Majounie E (2013). TREM2 variants in Alzheimer’s disease. N Engl J Med.

[24] Masuda T, Sankowski R, Staszewski O, Prinz M (2020). Microglia heterogeneity in the single-cell era. Cell Rep.

[25] Prinz M, Jung S, Priller J (2019). Microglia biology: one century of evolving concepts. Cell.

[26] Zhu C, Kros JM, van der Weiden M, Zheng P, Cheng C, Mustafa DAM (2017). Expression site of P2RY12 in residential microglial cells in astrocytomas correlates with M1 and M2 marker expression and tumor grade. Acta Neuropathol Commun.

[27] Cattaneo M (2015). P2Y12 receptors: structure and function. J Thromb Haemost.

[28] Davis J, Xu F, Deane R, Romanov G, Previti ML, Zeigler K (2004). Early-onset and robust cerebral microvascular accumulation of amyloid beta-protein in transgenic mice expressing low levels of a vasculotropic Dutch/Iowa mutant form of amyloid beta-protein precursor. J Biol Chem.

[29] Jäkel L, Van Nostrand WE, Nicoll JAR, Werring DJ, Verbeek MM (2017). Animal models of cerebral amyloid angiopathy. Clin Sci (Lond).

[30] Ronaldson PT, Davis TP. Regulation of blood–brain barrier integrity by microglia in health and disease: a therapeutic opportunity. J Cereb Blood Flow Metab. 2020. 10.1177/0271678X20951995. 10.1177/0271678X20951995 PMC7687032 32928017

[31] Korin B, Ben-Shaanan TL, Schiller M, Dubovik T, Azulay-Debby H, Boshnak NT (2017). High-dimensional, single-cell characterization of the brain’s immune compartment. Nat Neurosci.

[32] Popa-Wagner A, Petcu EB, Capitanescu B, Hermann DM, Radu E, Gresita A (2020). Ageing as a risk factor for cerebral ischemia: underlying mechanisms and therapy in animal models and in the clinic. Mech Ageing Dev.

[33] Hsiao K, Chapman P, Nilsen S, Eckman C, Harigaya Y, Younkin S (1996). Correlative memory deficits, Abeta elevation, and amyloid plaques in transgenic mice. Science.

[34] Saito S, Yamamoto Y, Maki T, Hattori Y, Ito H, Mizuno K (2017). Taxifolin inhibits amyloid-β oligomer formation and fully restores vascular integrity and memory in cerebral amyloid angiopathy. Acta Neuropathol Commun.

[35] Kokjohn TA, Roher AE (2009). Amyloid precursor protein transgenic mouse models and Alzheimer’s disease: understanding the paradigms, limitations, and contributions. Alzheimers Dement.

[36] Spychala MS, Venna VR, Jandzinski M, Doran SJ, Durgan DJ, Ganesh BP (2018). Age-related changes in the gut microbiota influence systemic inflammation and stroke outcome. Ann Neurol.

[37] Miao J, Xu F, Davis J, Otte-Höller I, Verbeek MM, Van Nostrand WE (2005). Cerebral microvascular amyloid beta protein deposition induces vascular degeneration and neuroinflammation in transgenic mice expressing human vasculotropic mutant amyloid beta precursor protein. Am J Pathol.

[38] Chauhan A, Moser H, McCullough LD (2017). Sex differences in ischaemic stroke: potential cellular mechanisms. Clin Sci (Lond).

[39] Verma R, Ritzel RM, Harris NM, Lee J, Kim T, Pandi G (2018). Inhibition of miR-141-3p ameliorates the negative effects of poststroke social isolation in aged mice. Stroke.

[40] Lee J, D’Aigle J, Atadja L, Quaicoe V, Honarpisheh P, Ganesh BP, et al. Gut microbiota-derived short-chain fatty acids promote post-stroke recovery in aged mice. Circ Res. 2020; Available from: http://www.ncbi.nlm.nih.gov/pubmed/32354259. 10.1161/CIRCRESAHA.119.316448 PMC7415518 32354259

[41] Lee J, d’Aigle J, Atadja L, Quaicoe V, Honarpisheh P, Ganesh BP, et al. Gut microbiota-derived short-chain fatty acids promote post-stroke recovery in aged mice. Circ Res. 2020. 10.1161/CIRCRESAHA.119.316448. 10.1161/CIRCRESAHA.119.316448 PMC7415518 32354259

[42] Chen H-R, Sun Y-Y, Chen C-W, Kuo Y-M, Kuan IS, Tiger Li Z-R (2020). Fate mapping via CCR2-CreER mice reveals monocyte-to-microglia transition in development and neonatal stroke. Sci Adv.

[43] Garner KM, Amin R, Johnson RW, Scarlett EJ, Burton MD (2018). Microglia priming by interleukin-6 signaling is enhanced in aged mice. J Neuroimmunol.

[44] Woollard KJ, Geissmann F (2010). Monocytes in atherosclerosis: subsets and functions. Nat Rev Cardiol.

[45] Tay TL, Sagar, Dautzenberg J, Grün D, Prinz M (2018). Unique microglia recovery population revealed by single-cell RNAseq following neurodegeneration. Acta Neuropathol Commun.

[46] Koellhoffer EC, McCullough LD, Ritzel RM. Old maids: aging and its impact on microglia function. Int J Mol Sci. 2017;18 Available from: http://www.ncbi.nlm.nih.gov/pubmed/28379162. 10.3390/ijms18040769 PMC5412353 28379162

[47] Yang H, Graham LC, Reagan AM, Grabowska WA, Schott WH, Howell GR (2019). Transcriptome profiling of brain myeloid cells revealed activation of Itgal, Trem1, and Spp1 in western diet-induced obesity. J Neuroinflammation.

[48] Bennett FC, Bennett ML, Yaqoob F, Mulinyawe SB, Grant GA, Hayden Gephart M (2018). A combination of ontogeny and CNS environment establishes microglial identity. Neuron.

[49] Grassivaro F, Menon R, Acquaviva M, Ottoboni L, Ruffini F, Bergamaschi A (2020). Convergence between microglia and peripheral macrophages phenotype during development and neuroinflammation. J Neurosci.

[50] Keren-Shaul H, Spinrad A, Weiner A, Matcovitch-Natan O, Dvir-Szternfeld R, Ulland TK (2017). A unique microglia type associated with restricting development of Alzheimer’s disease. Cell.

[51] Krasemann S, Madore C, Cialic R, Baufeld C, Calcagno N, El Fatimy R (2017). The TREM2-APOE pathway drives the transcriptional phenotype of dysfunctional microglia in neurodegenerative diseases. Immunity.

[52] Walker DG, Tang TM, Mendsaikhan A, Tooyama I, Serrano GE, Sue LI, et al. Patterns of expression of purinergic receptor P2RY12, a putative marker for non-activated microglia, in aged and Alzheimer’s disease brains. Int J Mol Sci. 2020;21 Available from: http://www.ncbi.nlm.nih.gov/pubmed/31968618. 10.3390/ijms21020678 PMC7014248 31968618

[53] Getts DR, Terry RL, Getts MT, Müller M, Rana S, Shrestha B (2008). Ly6c + “inflammatory monocytes” are microglial precursors recruited in a pathogenic manner in West Nile virus encephalitis. J Exp Med.

[54] Rangaraju S, Raza SA, Li NX, Betarbet R, Dammer EB, Duong D (2018). Differential phagocytic properties of CD45^low^ microglia and CD45^high^ brain mononuclear phagocytes-activation and age-related effects. Front Immunol.

[55] Sousa C, Golebiewska A, Poovathingal SK, Kaoma T, Pires-Afonso Y, Martina S, et al. Single-cell transcriptomics reveals distinct inflammation-induced microglia signatures. EMBO Rep. 2018;19 Available from: http://www.ncbi.nlm.nih.gov/pubmed/30206190. 10.15252/embr.201846171 PMC6216255 30206190

[56] Bedi SS, Smith P, Hetz RA, Xue H, Cox CS (2013). Immunomagnetic enrichment and flow cytometric characterization of mouse microglia. J Neurosci Methods.

[57] Stratoulias V, Venero JL, Tremblay M-È, Joseph B (2019). Microglial subtypes: diversity within the microglial community. EMBO J.

