# Supplementary figures and images for: Potential caveats of putative microglia-specific markers for assessment of age-related cerebrovascular neuroinflammation
Source: J Neuroinflammation. 2020 Dec 1;17:366. doi: 10.1186/s12974-020-02019-5 (PMC7709276; doi:10.1186/s12974-020-02019-5)

## Slide 1
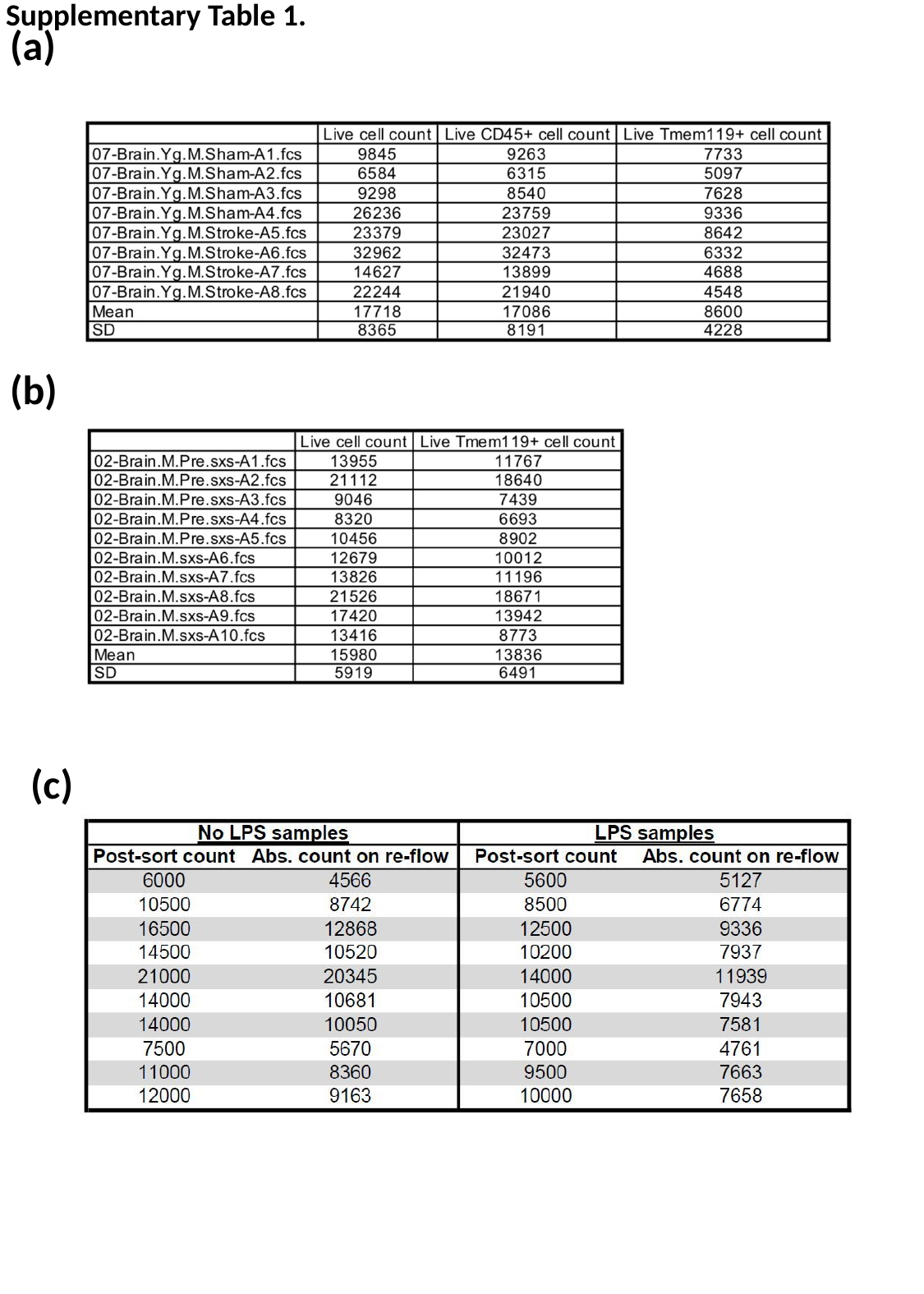

Supplementary Table 1.
(a)
(b)
(c)

Supplement: Supplementary file 1 — Additional file 1: Table S1. Absolute cell counts for brain flow preps from automated volumetric data obtained from cytometers (Cytoflex S for Main Figs. 1 and 2 and BD FACSMelody for Fig. 5) for MCAO (a), CAA (b), and ex-vivo LPS on sorted MG (c) experiments. [file 12974_2020_2019_MOESM1_ESM.pptx]
