# Supplementary material for: Potential caveats of putative microglia-specific markers for assessment of age-related cerebrovascular neuroinflammation
Source: J Neuroinflammation. 2020 Dec 1;17:366. doi: 10.1186/s12974-020-02019-5 (PMC7709276; doi:10.1186/s12974-020-02019-5)
Supplement: Supplementary file 2 — Additional file 2: Figure S1. Brain gating strategy for Live Tmem119+ cells using fluorescence minus one control for positive gating. Figure S2. Evaluation of Live Tmem119+ population for P2RY12 indicates that all Tmem119+ cells are P2RY12+, as well (top plots) and vice versa (bottom plots) (a). Within the CD45highCD11b+population, a small percentage of double positive for Tmem119 and P2RY12 were Ly6C+ while the Tmem119(-)P2RY12(-) were indeed predominantly Ly6C+, suggesting their peripheral origin (b). Figure S3. Blood Live cells from sham or stroke mice do not contain any significant Tmem119+P2RY12+ cells. [file 12974_2020_2019_MOESM2_ESM.pptx]

## Slide 1
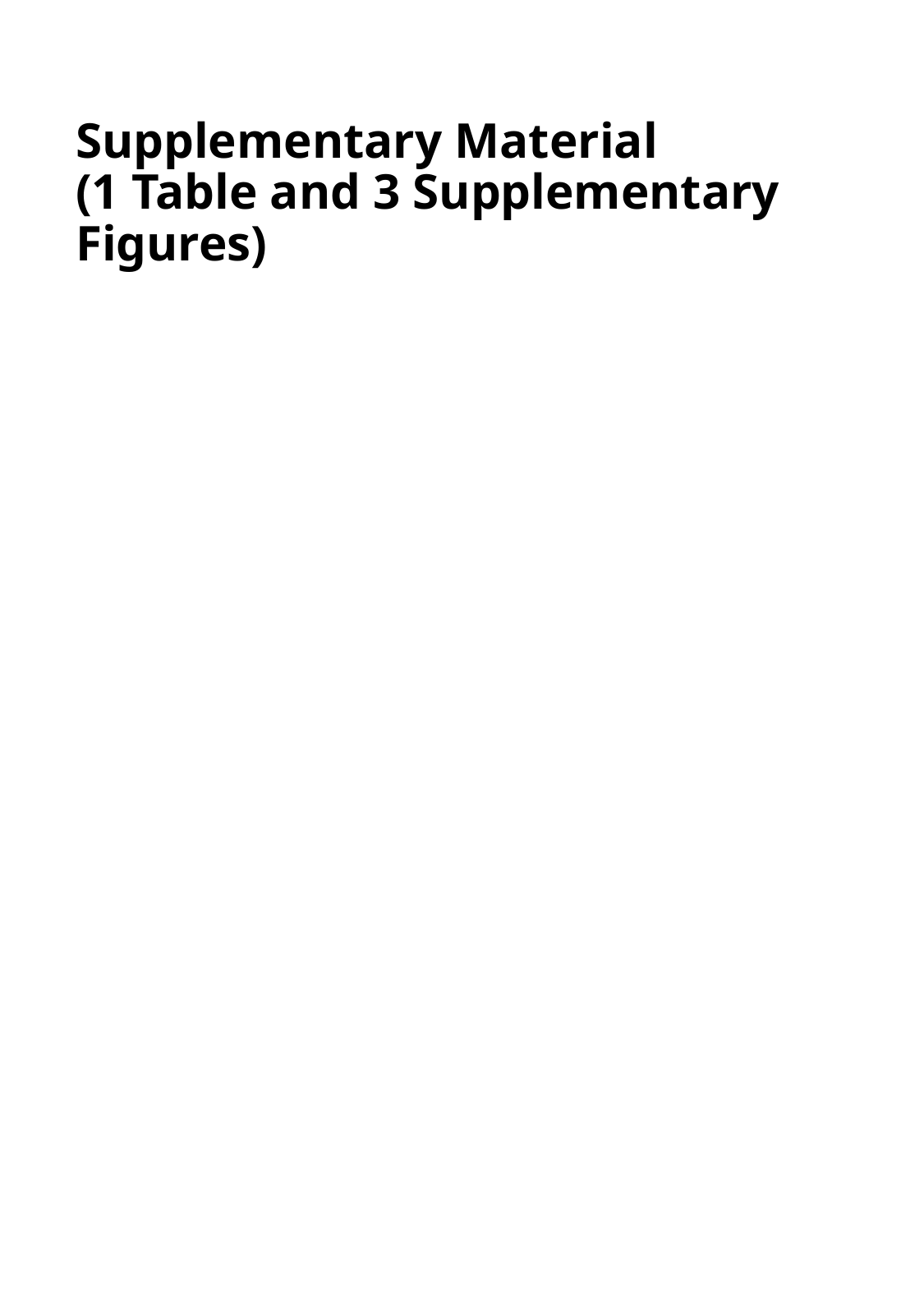

# Supplementary Material (1 Table and 3 Supplementary Figures)

## Slide 2
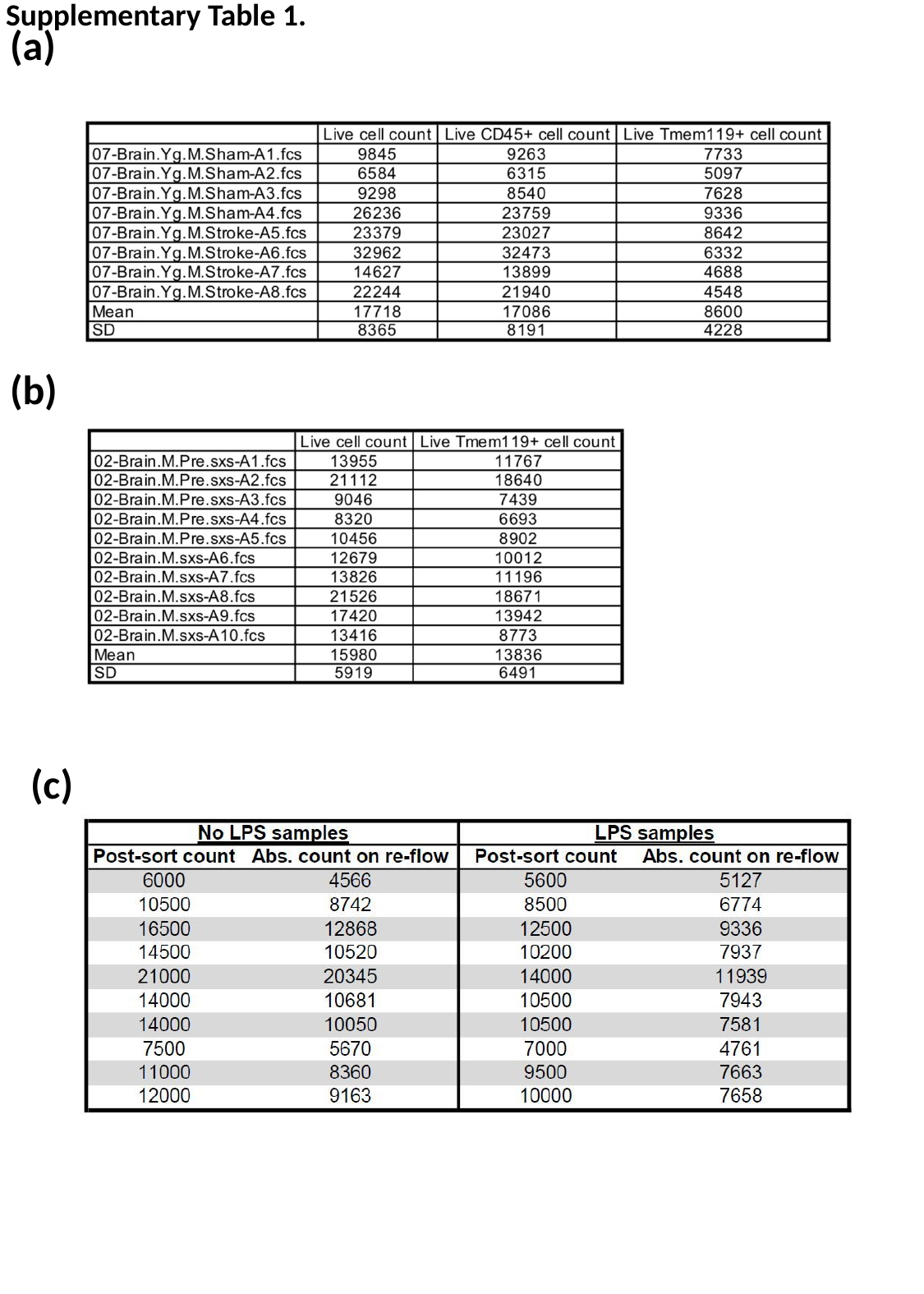

Supplementary Table 1.
(a)
(b)
(c)

## Slide 3
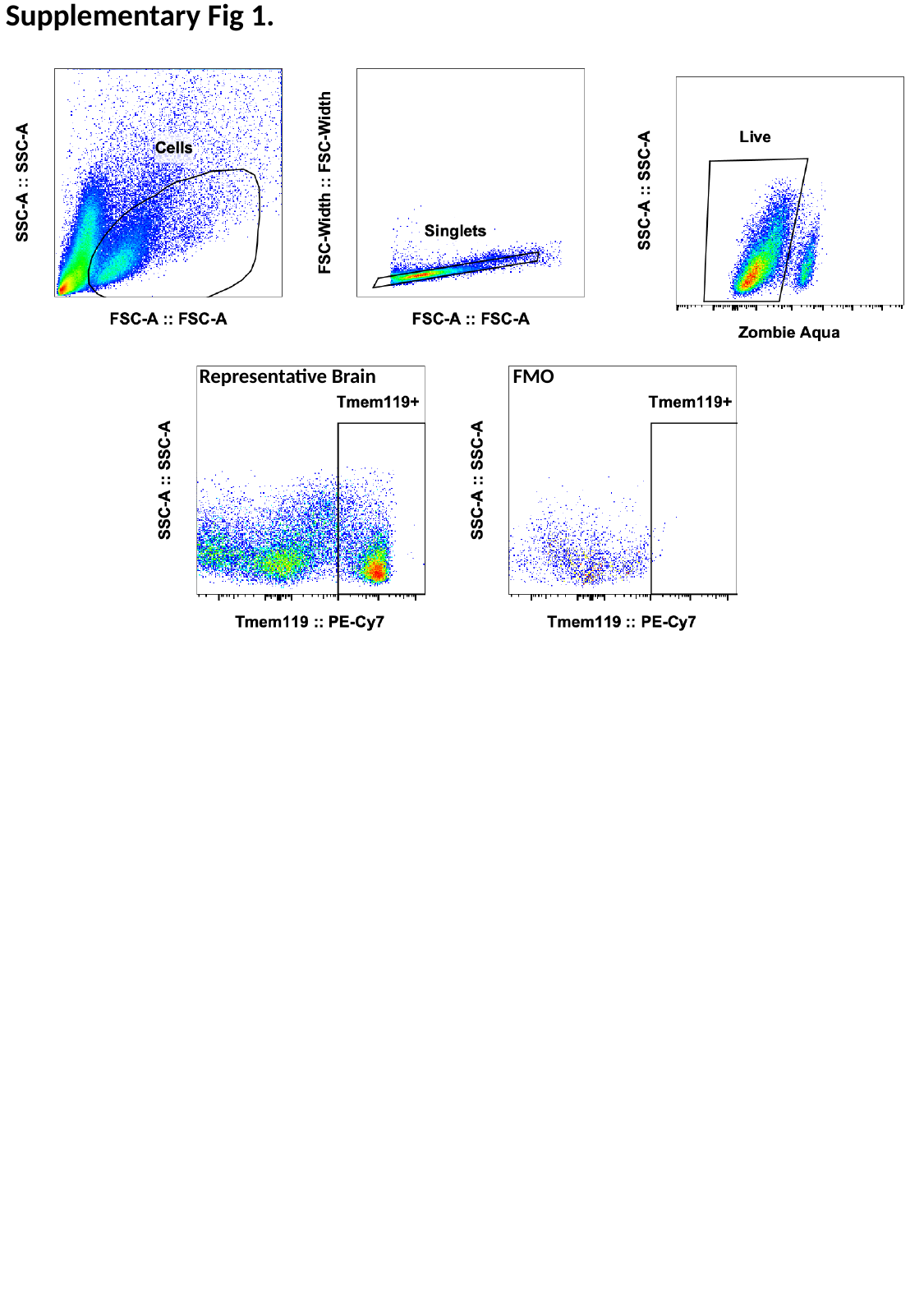

Supplementary Fig 1.
Representative Brain
FMO

## Slide 4
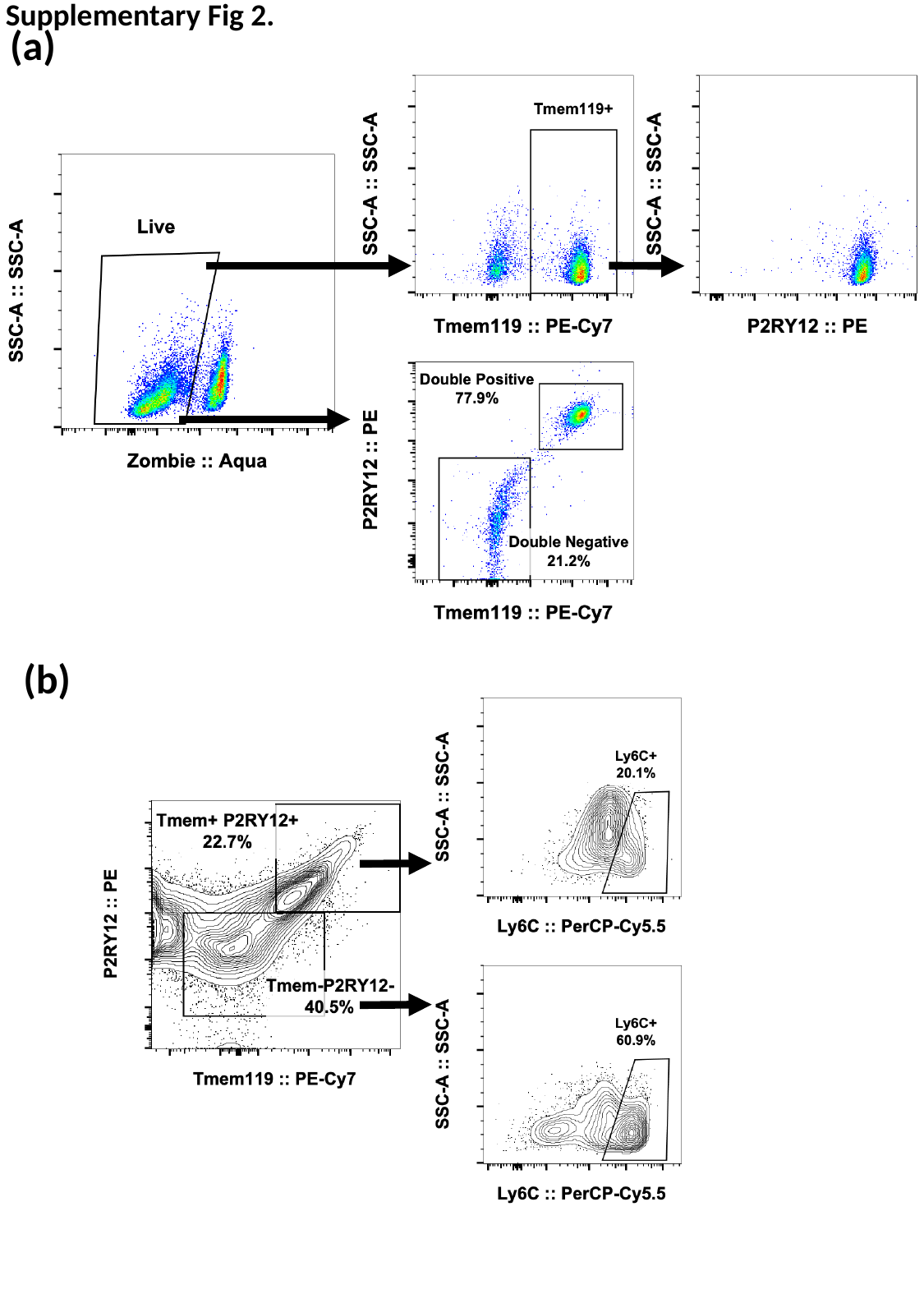

Supplementary Fig 2.
(a)
(b)

## Slide 5
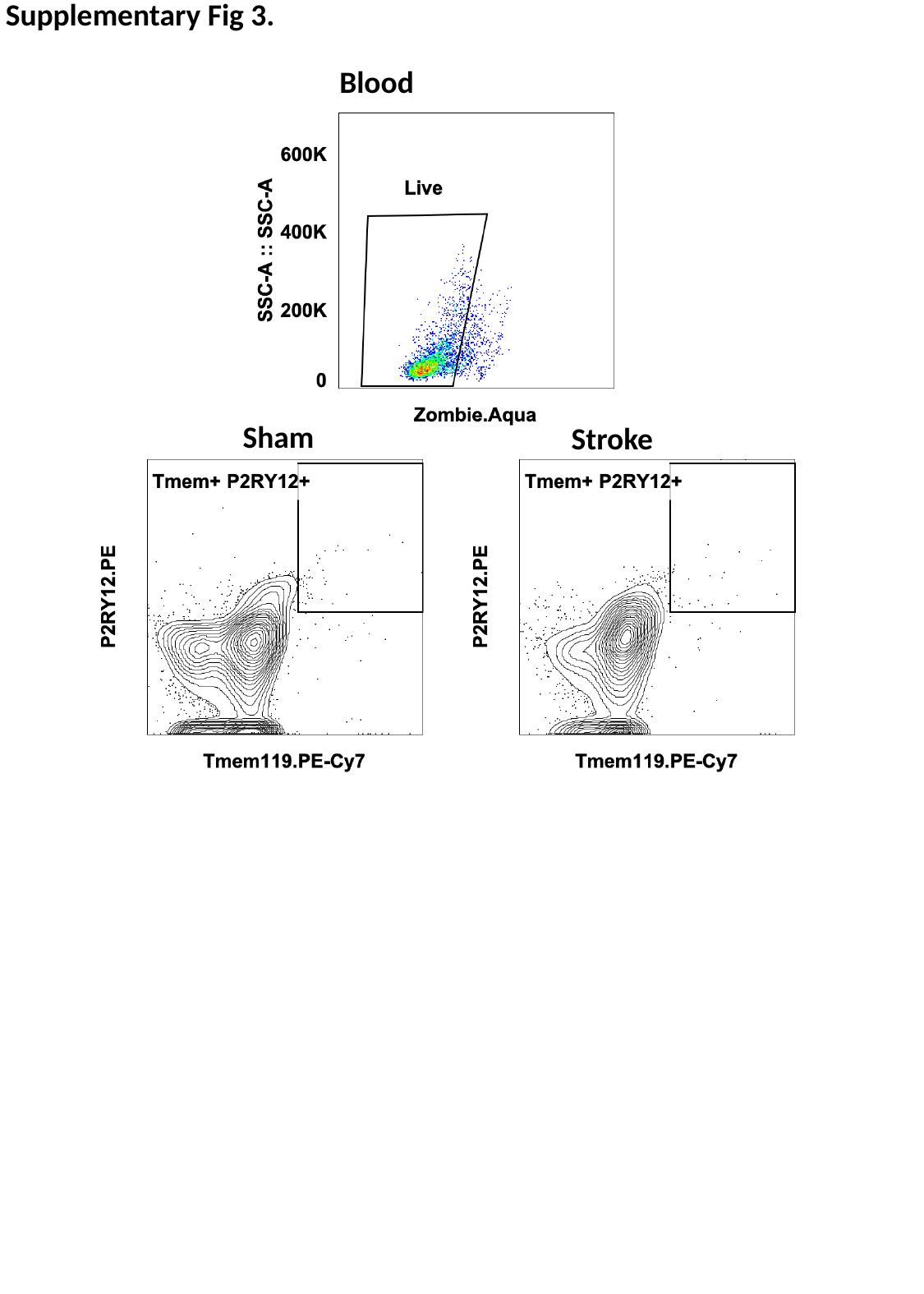

Supplementary Fig 3.
Blood
Sham
Stroke
